# *Helicobacter pylori* and oesophageal and gastric cancers in a prospective study in China

**DOI:** 10.1038/sj.bjc.6603517

**Published:** 2006-12-19

**Authors:** F Kamangar, Y-L Qiao, M J Blaser, X-D Sun, H Katki, J-H Fan, G I Perez-Perez, C C Abnet, P Zhao, S D Mark, P R Taylor, S M Dawsey

**Affiliations:** 1 Division of Cancer Epidemiology and Genetics, National Cancer Institute, 6120 Executive Blvd, Rm 3034, Bethesda, MD 20892-7232, USA; 2 Department of Cancer Epidemiology, Cancer Institute of the Chinese Academy of Medical Sciences, PO Box 2258, Beijing, 100021 China; 3 Department of Medicine, New York University School of Medicine, New York, NY, USA; 4 Department of Microbiology, New York University School of Medicine, New York, NY, USA; 5 Department of Preventive Medicine and Biometrics, University of Colorado Health Sciences Center, Denver, CO, USA

**Keywords:** *Helicobacter pylori*, oesophageal cancer, gastric cancer, China

## Abstract

In a cohort of 29 584 residents of Linxian, China, followed from 1985 to 2001, we conducted a case–cohort study of the magnitude of the association of *Helicobacter pylori* seropositivity with cancer risk in a random sample of 300 oesophageal squamous cell carcinomas, 600 gastric cardia adenocarcinomas, all 363 diagnosed gastric non-cardia adenocarcinomas, and a random sample of the entire cohort (*N*=1050). Baseline serum was evaluated for IgG antibodies to whole-cell and CagA *H. pylori* antigens by enzyme-linked immunosorbent assay. Risks of both gastric cardia and non-cardia cancers were increased in individuals exposed to *H. pylori* (Hazard ratios (HRs) and 95% confidence intervals=1.64; 1.26–2.14, and 1.60; 1.15–2.21, respectively), whereas risk of oesophageal squamous cell cancer was not affected (1.17; 0.88–1.57). For both cardia and non-cardia cancers, HRs were higher in younger individuals. With longer time between serum collection to cancer diagnosis, associations became stronger for cardia cancers but weaker for non-cardia cancers. CagA positivity did not modify these associations. The associations between *H. pylori* exposure and gastric cardia and non-cardia adenocarcinoma development were equally strong, in contrast to Western countries, perhaps due to the absence of Barrett's oesophagus and oesophageal adenocarcinomas in Linxian, making all cardia tumours of gastric origin, rather than a mixture of gastric and oesophageal malignancies.

*Helicobacter pylori* is an important risk factor for gastric non-cardia adenocarcinoma (Nomura *et al*, 2005; Kamangar *et al*, 2006), but its association with gastric cardia adenocarcinoma is less clear. A combined analysis of 12 prospective studies (Helicobacter and Cancer Collaborative Group, 2001) showed an overall null association between *H. pylori* and gastric cardia cancer risk. However, significant geographic variation may exist: most East-Asian studies have shown an increased risk, whereas most Western studies have shown either a null association or reduced risk (Dawsey *et al*, 2002). For both cardia and non-cardia cancers, the risk estimates may also be a function of study design and length of follow-up (Helicobacter and Cancer Collaborative Group, 2001), age at diagnosis (Kato *et al*, 2004), and *H. pylori* strain (Blaser *et al*, 1995).

Very few studies have examined the association between *H. pylori* infection and oesophageal squamous cell carcinoma. The results of these studies have been inconsistent, and both positive (Ye *et al*, 2004) and inverse associations (Siman *et al*, 2001; Wu *et al*, 2005) have been reported.

People of Linxian, China, have very high rates of oesophageal squamous cell carcinoma and gastric cardia adenocarcinoma, and also moderately high rates of gastric non-cardia adenocarcinoma; approximately 20% of residents die of these cancers. We conducted this long-term prospective case–cohort study to estimate the magnitude of the association of *H. pylori* seropositivity and risk of these cancers in Linxian, and to examine these associations by time from serum collection to cancer diagnosis, age, and CagA positivity.

## MATERIALS AND METHODS

### Study cohort

Subjects were selected from the cohort of all participants in the Linxian General Population Trial, which is described elsewhere (Blot *et al*, 1993; Li *et al*, 1993). In brief, the participants were 29 584 healthy adults aged 40–69 years from four Linxian communes. In the spring of 1985, 1 year before the start of intervention, each participant was interviewed, given a brief physical examination, and had 10 ml of blood drawn. After collection, serum specimens were separated, aliquoted, and stored frozen at −70°C for future analyses. All participants were randomly assigned to one of eight vitamin/mineral combinations, and the supplements were distributed from March 1986 to May 1991. Throughout the trial period, local health care providers recorded cancer incidence and mortality data at monthly intervals. Periodic surveys were conducted to verify completeness and accuracy of the medical information. Pathology slides and/or X-rays were available for 85% of the cancer cases in this study, and these were reviewed by a panel of American and Chinese experts. For cancer cases without such diagnostic materials and for deaths due to causes other than cancer, reviews were performed by senior Chinese experts. In the subsequent 10 years post-trial, subjects were contacted monthly, either by village health workers or by study interviewers, and cancer diagnoses were verified by senior Chinese diagnosticians from Beijing. Case ascertainment is considered complete and loss to follow-up minimal (*n*=176 or <1%). Outcomes for the present study were based on follow-up data through May 2001.

For anatomic localisation of gastric tumours, cancers were defined as cardia cancers if they were in the most proximal 3 cm of the stomach, and non-cardia cancers if they were distal to this region (Blot *et al*, 1993; Li *et al*, 1993). Ninety-five percent of anatomic localisations were made using endoscopy, surgery, and/or X-rays.

The trial was approved by the institutional review boards of the Cancer Institute of Chinese Academy of Medical Sciences and the US National Cancer Institute.

### Cases and subcohort

By March 2001, 1958 cases of oesophageal squamous cell carcinoma, 1089 cases of gastric cardia adenocarcinoma, and 363 cases of gastric non-cardia adenocarcinoma were diagnosed. There were no cases of oesophageal adenocarcinoma. For the *H. pylori* assays, we used a case–cohort design, selecting a random sample of 300 oesophageal cancers, 600 cardia cancers, and all 363 non-cardia cancers as case subjects. For the comparison group, we selected a random sample of 1050 subjects from the entire baseline cohort, hereafter called the subcohort. Some of the subcohort subsequently developed cancer, and these were added to the case group from the date of diagnosis (and correspondingly removed from this date from the subcohort). Serum samples were available for 2138 (95%) of subjects. The final numbers were 335 oesophageal cancer cases (271 selected cases and 64 subcohort cases), 582 cardia cancer cases (549 selected cases and 33 subcohort cases), 343 non-cardia cancer cases (326 selected cases and 17 subcohort cases), and 992 subcohort members (with or without one of these cancers).

### Serologic assays

Serum was evaluated for IgG antibodies to whole-cell (WC) and CagA *H. pylori* antigens by enzyme-linked immunosorbent assay, as described previously (Limburg *et al*, 2001). Similar to previous studies (Limburg *et al*, 2001), seropositivity cutpoints were defined as optical density ratios ⩾1.0 for WC antibodies and ⩾0.35 for CagA antibodies. Individuals who were negative for both antibodies (WC−, CagA−) were classified as *H. pylori* seronegative, whereas individuals who were seropositive for either WC or CagA antibodies were classified as *H. pylori* seropositive. This classification system was selected because culture-based studies have shown that individuals who are negative for *H. pylori* WC antibodies but positive for CagA antibodies are true positives (Romero-Gallo *et al*, 2002). Seropositive individuals were further classified as carrying CagA-negative strains (WC+, CagA−) or CagA-positive strains (WC+ or WC−, CagA+) (Romero-Gallo *et al*, 2002).

Experienced technicians who were unaware of subjects' case–control status performed the serologic assays in duplicate. When two assayed aliquots provided indeterminate results (i.e., the values straddled the seropositivity threshold), additional aliquots were analysed and the average of all results (excluding obvious outliers) was used to determine serologic status. One hundred and twelve external quality control serum samples, aliquoted from a single large pooled serum sample from Linxian, were equally distributed among 56 different batches. On the basis of these samples, the coefficients of variation were 15 and 20% for the WC and CagA assays, respectively.

### Statistical methods

Consistent with previous studies of upper gastrointestinal cancers in Linxian, we considered age (years), sex (male *vs* female), history of smoking and alcohol consumption (yes *vs* no), and body mass index (kg m^−2^) as potential confounders. Mean and standard deviation of continuous variables (age and body mass index), and numbers and percentages of categorical variables (sex, history of smoking and alcohol consumption) were calculated and reported for the subcohort and each cancer type.

We used Cox proportional hazards models to estimate the crude and adjusted hazard ratios (HRs) and 95% confidence intervals (95% CIs) for the associations with *H. pylori*, using special R-software (Mark and Katki, 2006). As potential confounders made no material difference in the estimated HRs, models were only adjusted for age and sex. Hazard ratios were calculated for each cancer, and also by time from serum collection to diagnosis (⩽5, 5.1–10, and >10 years), age group (⩽55 and >55 years at study entry), and *H. pylori* strain (CagA-positive *vs* CagA-negative). The assumption of proportional hazards was explored by calculating HRs in three strata of time from serum collection to diagnosis (⩽5, 5.1–10, and >10 years). These hazards changed significantly over time for cardia and noncardia cancers, and were therefore reported separately for each time period. All analyses were repeated using logistic regression models and similar results were obtained (data not shown). Throughout the paper, all *P*-values are two-sided and *P*-values ⩽0.05 were considered as significant.

## RESULTS

Of the 992 subcohort members, 487 (49%) were positive for antibodies to both *H. pylori* WC and CagA antigens, 175 (18%) for only WC antibodies, 65 (7%) for only CagA antibodies, and 265 (27%) for neither. Thus, 73% of the subcohort members had serologic evidence of *H. pylori* exposure.

Table 1 shows the demographic characteristics and potential confounders in cancer cases and subcohort members. Compared to the subcohort, all subgroups of cancer cases were older, and cardia and non-cardia cancer cases had a higher proportion of males. Prevalence of tobacco smoking, alcohol drinking, and mean body mass index were similar among all subgroups (Table 1), and their frequency by case status similar to those for the whole cohort (Tran *et al*, 2005).

In all, 73% of the subcohort, 76% of oesophageal, 81% of cardia, and 80% of the non-cardia cancer cases were positive for serum *H. pylori* antibodies (Table 2). Adjusted HRs (95% CI) were 1.17 (0.88–1.57) for oesophageal, 1.64 (1.26–2.14) for cardia, and 1.60 (1.15–2.21) for non-cardia cancers.

Oesophageal cancer risk estimates did not show a clear trend with time from serum collection, and none was significant (Table 3), HRs (95% CIs) being 1.43 (0.90–2.29), 0.78 (0.50–1.20), and 1.21 (0.74–1.96) for cases diagnosed ⩽5, 5.1–10, and >10 years after serum collection, respectively (Table 3). The association between *H. pylori* and gastric cardia cancer was stronger in cases diagnosed >10 years after serum collection than earlier: HRs (95% CIs) were 1.30 (0.92–1.85), 1.35 (0.93–1.97), and 2.41 (1.50–3.88) for cardia cancer cases diagnosed in the corresponding periods (Table 3). For non-cardia cancer, the risk estimates declined after the first 5 years of follow-up, being 2.32 (1.35–3.97), 1.23 (0.77–1.98) and 1.27 (0.78–2.08), respectively (Table 3).

Anti-*H. pylori* seropositivity was not a risk factor for oesophageal squamous cell cancer in individuals ⩽55 or those >55 years of age at diagnosis (Table 3). The associations between serum antibodies to *H. pylori* and risk of both cardia and non-cardia cancers were nonsignificantly stronger for individuals ⩽55 years of age at diagnosis; HRs were close to 2.0 for those ⩽55 years, but approximately 1.4 for those >55 at diagnosis (Table 3).

Table 4 shows the crude and adjusted HRs (95% CIs) for the associations between CagA-negative and CagA-positive strains of *H. pylori* and each cancer type. For oesophageal cancer, the HR point estimates were less strong for CagA-positive than CagA-negative strains (adjusted HR 1.08 and 1.43, respectively). For cardia cancers, the adjusted HRs were slightly stronger for CagA-positive than CagA-negative strains (1.75 and 1.35, respectively), although their difference was not statistically significant. For non-cardia cancers, the HR point estimates were similar (≈1.60) for CagA-positive and CagA-negative strains.

## DISCUSSION

This study showed a positive association (HR 1.6) between *H. pylori* infection and risk of gastric non-cardia cancer in Linxian, lower than the average found in meta-analyses either from international data (RR 3) (Huang *et al*, 1998; Eslick *et al*, 1999; Helicobacter and Cancer Collaborative Group, 2001) or from Chinese studies alone (RR 3) (Xue *et al*, 2001). The smaller risk observed in Linxian may reflect the unusual predominance of other risk factors in this population. Nutritional deficiency is common in Linxian, and supplementation with a combination of selenium, vitamin E, and beta-carotene has been shown to reduce the risk of gastric cancer mortality in this area (Blot *et al*, 1993). If a large proportion of non-cardia cancers in Linxian are caused by other factors, then the relative risk for the association with *H. pylori* will be attenuated. A similar argument may explain the stronger associations seen in younger individuals in this and certain other studies (Nomura *et al*, 1991; Hansson *et al*, 1993); older people may have other strong risk factors for non-cardia cancer.

The weaker *H. pylori* association with non-cardia cancer cases diagnosed more than 5 years after serum collection differed from that in cardia cancer cases, and also from that in most other long-term prospective studies (Helicobacter and Cancer Collaborative Group, 2001).

CagA-positive strains of *H. pylori* may be associated with a greater risk of gastric non-cardia cancer by further increasing the turnover of the gastric epithelium (Hatakeyama, 2004). Some but not all studies have found that CagA-positive strains of *H. pylori* are associated with a higher risk of non-cardia adenocarcinoma than CagA-negative strains, and a meta-analysis of these studies found that the presence of CagA-positive strains increased this risk by two-fold (Huang *et al*, 2003). In the current study, CagA-positive strains were not associated with a further increased risk of non-cardia cancer.

The strength of the association with *H. pylori* infection was similar for gastric cardia (HR 1.6) as for non-cardia gastric cancer, as in several other East-Asian studies, but unlike in Western countries, where the association with cardia cancer is null or inverse (Hansen *et al*, 1999; Kamangar *et al*, 2006). This may reflect a different definition of cardia cancer in Western *vs* Eastern countries (Dawsey *et al*, 2002). In Western countries, where Barrett's oesophagus and adenocarcinoma of the oesophagus are common, cardia cancer most likely includes a mixture of gastric cardia and oesophageal adenocarcinomas. The cardia of the stomach is a very small area, and differentiating large adenocarcinomas arising in the gastric cardia from those arising in the lower oesophagus or the body of the stomach is not always possible. In Linxian, however, Barrett's oesophagus and oesophageal adenocarcinoma are very rare or non-existent, so cardia tumours probably mainly arise in the proximal stomach. This is supported by our clinical experience in this population, in which all small asymptomatic adenocarcinomas discovered near the gastroesophageal junction were on the gastric side of this junction. The studies of *H. pylori* and gastric cardia cancer reported from Western countries and East Asia suggest overall that *H. pylori* is probably a risk factor for adenocarcinomas in the proximal stomach but is probably protective against those in the distal oesophagus.

The point estimates for the association between *H. pylori* and cardia cancer were stronger in younger individuals, for cases that were diagnosed long after serum collection, and for CagA-positive strains. We did not find an association between *H. pylori* and oesophageal squamous cell cancer, irrespective of age, time from serum collection to diagnosis, or CagA-positivity. *H. pylori* does not live in the oesophagus, but *H. pylori*-induced gastric atrophy and subsequently reduced gastric acidity may allow overgrowth of bacteria, which produce nitrosamines and thereby increase oesophageal cancer risk (Ye *et al*, 2004). Both decreased (Siman *et al*, 2001; Wu *et al*, 2005) and increased (Ye *et al*, 2004) risks of oesophageal squamous cell cancer associated with *H. pylori* have been reported. A case–control study (128 cases) in Taiwan found an inverse association (adjusted odds ratio=0.51) (Wu *et al*, 2005), as did a Swedish prospective study (29 cases) (odds ratio=0.41) (Siman *et al*, 2001). In contrast, a population-based Swedish case–control study (85 cases) found a significant two to three fold increased risk associated with CagA-positive serotypes, and no association with CagA-negative serotypes (Ye *et al*, 2004).

In our data, smoking and alcohol consumption had only a small role in the aetiology of oesophageal squamous cell cancer. These two exposures are major risk factors for oesophageal squamous cell carcinoma in Western populations (Brown *et al*, 1994, 2001), but play a much smaller role in such high-risk areas, such as Linxian (Tran *et al*, 2005) or northeastern Iran (Cook-Mozaffari *et al*, 1979; Islami *et al*, 2004).

The strengths of this study include its large sample size, prospective design, long-term follow-up, availability of data on tumour location, the presence of data on potential confounders, and measurement of both WC and CagA antibodies. This is the largest reported prospective study of *H. pylori* and risk of these three cancers. Previous studies of *H. pylori* and cardia cancer have only had 20–60 cases, and may have included some oesophageal adenocarcinomas misclassified as cardia cancers.

## Figures and Tables

**Table 1:** Demographic characteristics, tobacco use, and alcohol consumption among cases and the subcohort

	**Subcohort (*n*=992)**	**ESCC (*n*=335)**	**GCA (*n*=582)**	**GNCA (*n*=343)**
Mean age in years(s.d.)	51.9 (8.9)	54.5 (8.5)	55.5 (7.7)	56.0 (7.9)
Number of males (%)	449 (45.3)	155 (46.3)	351 (60.3)	228 (66.5)
Number of male smokers (%)^a^	329 (73.6)	111 (71.6)	237 (68.1)	161 (70.6)
Number of alcohol consumers (%)^a^	239 (24.1)	68 (20.3)	138 (23.4)	81 (23.6)
Body mass index (kg m^−2^) (s.d.)	22.0 (2.5)	21.5 (2.4)	21.8 (2.3)	21.5 (2.2)

a Smoking and drinking were both categorized as binary variables. Smoking in this population was almost entirely limited to male subjects, so the numbers and percents were calculated only for men. Male subjects who ever smoked cigarettes for six or more months were classified as smokers; subjects who drank any alcoholic beverage in the last 12 months were classified as alcohol consumers.

**Table 2:** Hazard ratios associated with anti-*H. pylori* seropositivity by anatomic subsite

	**Number tested**	**Number positive (%)**	**Crude HR (95% CI)**	**Adjusted HR^a^ (95% CI)**
Subcohort	992	727 (73)	—	—
Oesophageal squamous cell cancer	335	254 (76)	1.16 (0.88–1.55)	1.17 (0.88–1.57)
Gastric cardia cancer	582	473 (81)	1.59 (1.24–2.05)	1.64 (1.26–2.14)
Gastric non-cardia cancer	343	276 (80)	1.51 (1.12–2.05)	1.60 (1.15–2.21)

a Models were adjusted for age, age-squared, and sex.

**Table 3:** Hazard ratios associated with anti-*H. pylori* seropositivity by time to diagnosis and age

**Site**	**Time to diagnosis (years)**	**Age**	**Number tested**	**Number positive (%)**	**Adjusted HR^a^ (95% CI)**
Subcohort	—	—	992	727 (73)	—
Oesophageal squamous cell cancer	⩽5		124	101 (81)	1.43 (0.90–2.29)
	5.1–10		108	75 (69)	0.78 (0.50–1.20)
	>10		103	78 (76)	1.21 (0.74–1.96)
		⩽55	166	129 (78)	1.29 (0.86–1.94)
		>55	169	125 (74)	1.10 (0.73–1.66)
					
Gastric cardia cancer					
	⩽5		216	171 (79)	1.30 (0.92–1.85)
	5.1–10		199	158 (79)	1.35 (0.93–1.97)
	>10		167	144 (86)	2.41 (1.50–3.88)
		⩽55	287	244 (85)	2.04 (1.38–3.01)
		>55	295	229 (78)	1.39 (0.96–2.01)
					
Gastric non-cardia cancer					
	⩽5		124	107 (86)	2.32 (1.35–3.97)
	5.1–10		113	88 (78)	1.23 (0.77–1.98)
	>10		106	81 (76)	1.27 (0.78–2.08)
		⩽55	151	128 (85)	2.06 (1.25–3.40)
		>55	192	148 (77)	1.36 (0.88–2.10)

a Models were adjusted for age, age-squared, and sex.

**Table 4:** Hazard ratios associated with Cag-positive and Cag-negative *H. pylori*

**Site**	**Hp status**	***N* (%)**	**Crude HR (95% CI)**	**Adjusted HR^a^ (95% CI)**
Subcohort	Negative	265 (27)	—	—
	Cag-negative	175 (18)	—	—
	Cag-positive	552 (56)	—	—
				
Oesophageal squamous cell cancer	Negative	81 (24)	—	—
	Cag-negative	76 (23)	1.49 (1.04–2.13)	1.43 (0.99–2.07)
	Cag-positive	178 (53)	1.07 (0.79–1.43)	1.08 (0.80–1.47)
				
Gastric cardia cancer	Negative	109 (19)	—	—
	Cag-negative	100 (17)	1.43 (1.03–2.00)	1.35 (0.95–1.92)
	Cag-positive	373 (64)	1.64 (1.27–2.13)	1.75 (1.32–2.30)
				
Gastric non-cardia cancer	Negative	67 (20)	—	—
	Cag-negative	74 (22)	1.73 (1.18–2.54)	1.62 (1.08–2.45)
	Cag-positive	202 (59)	1.45 (1.06–1.98)	1.58 (1.13–2.22)

Due to rounding, some percentages do not add up to 100%.

a Models were adjusted for age, age-squared, and sex.
